# Hidden Markov Model based stride segmentation on unsupervised free-living gait data in Parkinson’s disease patients

**DOI:** 10.1186/s12984-021-00883-7

**Published:** 2021-06-03

**Authors:** Nils Roth, Arne Küderle, Martin Ullrich, Till Gladow, Franz Marxreiter, Jochen Klucken, Bjoern M. Eskofier, Felix Kluge

**Affiliations:** 1 Machine Learning and Data Analytics Lab, Department of Artificial Intelligence in Biomedical Engineering, Friedrich-Alexander-Universität Erlangen-Nürnberg (FAU), Erlangen, Germany; 2 Department of Molecular Neurology, University Hospital of Erlangen, Erlangen, Germany

**Keywords:** HMM, IMU, Machine learning, Mobile gait analysis, Stride borders, Wearable sensors

## Abstract

**Background:**

To objectively assess a patient’s gait, a robust identification of stride borders is one of the first steps in inertial sensor-based mobile gait analysis pipelines. While many different methods for stride segmentation have been presented in the literature, an out-of-lab evaluation of respective algorithms on free-living gait is still missing.

**Method:**

To address this issue, we present a comprehensive free-living evaluation dataset, including 146.574 semi-automatic labeled strides of 28 Parkinson’s Disease patients. This dataset was used to evaluate the segmentation performance of a new Hidden Markov Model (HMM) based stride segmentation approach compared to an available dynamic time warping (DTW) based method.

**Results:**

The proposed HMM achieved a mean F1-score of 92.1% and outperformed the DTW approach significantly. Further analysis revealed a dependency of segmentation performance to the number of strides within respective walking bouts. Shorter bouts ($< 30$ strides) resulted in worse performance, which could be related to more heterogeneous gait and an increased diversity of different stride types in short free-living walking bouts. In contrast, the HMM reached F1-scores of more than 96.2% for longer bouts ($> 50$ strides). Furthermore, we showed that an HMM, which was trained on at-lab data only, could be transferred to a free-living context with a negligible decrease in performance.

**Conclusion:**

The generalizability of the proposed HMM is a promising feature, as fully labeled free-living training data might not be available for many applications. To the best of our knowledge, this is the first evaluation of stride segmentation performance on a large scale free-living dataset. Our proposed HMM-based approach was able to address the increased complexity of free-living gait data, and thus will help to enable a robust assessment of stride parameters in future free-living gait analysis applications.

**Supplementary Information:**

The online version contains supplementary material available at 10.1186/s12984-021-00883-7.

## Background

In typical clinical gait analysis settings, a physician or clinical expert observes a patient’s gait in a hospital hallway to assess, for example, postural instability or motor function, which are major symptoms of the neurodegenerative Parkinson’s Disease (PD) and strongly related to independence, quality of life or risk of falls [1]. Therefore, new tools and technologies to quantitatively and objectively assess various gait-related parameters are of increased interest to understand pathophysiological mechanisms, investigate treatment effects or quantify the disease state in PD [2]. Especially wearable sensors and, in particular, inertial measurement units (IMUs) are often used in clinical applications to measure spatio-temporal gait parameters in order to objectively monitor disease progression or fluctuations [3, 4].

Due to advancements in size, energy consumption, and usability of wearable sensors, mobile gait analysis systems are getting started to be used beyond the borders of laboratory assessments in home-monitoring as well as free-living scenarios [5, 6]. Here, mobile gait analysis solutions enable a continuous and detailed insight into a patient’s mobility and motor performance under more natural and realistic conditions compared to clinical snapshot assessments [5, 6].

However, independent of the recording environment, a robust segmentation of individual strides from the continuous sensor data is one of the first steps in most wearable gait analysis systems and a crucial part of the underlying signal processing pipeline [7]. Various different approaches have been proposed in the literature to solve the problem of stride segmentation for clinical gait analysis applications [8–22]. Methods vary with sensor location, ranging from the upper body like wrist, chest or lower back [9, 10] to the lower body with sensors attached to ankles or feet [11–15, 17–22] as well as with sensor modalities like IMUs or pressure sensors [7]. Peak detection [8, 9], wavelet-based approaches [10–13], template matching [14, 15] as well as probabilistic machine learning models like the Hidden Markov Model (HMM) [16–20], and deep learning approaches like convolutional neural networks [21, 22] have been successfully applied in supervised laboratory conditions.

Although all of the aforementioned studies obtained good results, they were constrained in most cases by a controlled and supervised laboratory setting. Furthermore, most studies only provide a limited amount of heterogeneity within the dataset due to the inclusion of only healthy subjects, a rather small number of strides, or evaluation on standardized gait tests.

In contrast, a continuous full day free-living recording contains a variety of different unstructured activities as well as different stride types like initiation, termination, turnings, or transitions between activities. Influences of the external environment like context, location, different undergrounds, or cognitive challenges are known to be confounding factors for certain gait parameters [6, 23]. For PD patients, day-to-day and intra-day fluctuations of motor symptoms (related to health and medication state) increase the heterogeneity and irregularity of strides even further [1].

Recent studies already highlighted the differences between laboratory and free-living gait analysis [24–26], but a technical evaluation of gait analysis pipelines on free-living data is still an open challenge. A major issue is the lack of free-living evaluation datasets, including ground truth information. For this reason, evaluation studies usually focus on at-lab recordings where for example pressurized mats [19] or motion capture systems [27] are used as gold-standard references. However, such system cannot be used for fully unconstrained free-living studies.

Martindale et al. [20] tried to address this issue by collecting data of several semi-realistic scenarios covering different activities with providing stride level annotations of a large cohort of 80 healthy subjects. They used video recordings combined with pressure insoles and a smart annotation approach to generate labels for all activities. However, the study still followed a supervised protocol with scripted scenarios and only healthy subjects.

Hickey et al. [28] also emphasized the lack of evaluation of gait analysis algorithms on free-living data. To address this issue, they equipped ten young and healthy subjects with a waist-mounted accelerometer together with a body-mounted camera filming the subject’s feet on two consecutive days for one hour each. Subjects could then perform free-living activities in a completely unsupervised setting, while the video streams were used to generate reference labels. However, Hickey et al. only evaluated macro gait parameters like walking bout duration and step counts, but did not investigate actual stride border locations.

To address this lack of missing evaluation of stride segmentation algorithms on free-living data, we present a fully unconstrained and unsupervised free-living dataset including stride border annotations of 146.574 single strides of a cohort of 28 PD patients. Due to stigmatization and privacy concerns, we decided to not use body-worn cameras or similar reference systems but opted for a semi-automated manual labeling approach of the IMU data itself. For this semi-automated labeling approach, an existing template matching implementation based on subsequent Dynamic Time Warping (DTW), presented by Barth et al. [14], was combined with extensive manual post-processing.

Furthermore, a new HMM-based segmentation approach was implemented for evaluation, as a probabilistic model is a promising candidate to be robust and at the same time flexible enough to handle the expected variety and heterogeneity within free-living patient data. Haji Ghassemi et al. [29] compared different stride segmentation approaches for PD patients and found that an HMM-based segmentation could outperform template matching methods like DTW or simple peak detection methods, reaching F1-Scores above 95% in laboratory settings. Additionally, HMMs showed the potential to handle also classification or sub-phase segmentation tasks. Martindale et al. [30] and Mannini et al. [19] presented HMMs, which can be applied beyond pure stride segmentation for gait phase segmentation and gait event detection by modelling swing and stance phase by individual models. Panahandeh et al. [31] used a combination of multiple individual HMMs for activity classification based on a cycle level identification. Furthermore, HMMs were successfully applied for at-lab gait analysis in hereditary spastic paraplegia (HSP) patients, where the heterogeneity of the disease required the need of personalized models and proofed their applicability in segmentation of severely impaired and strongly heterogeneous gait [30].

Although the first steps towards the development and evaluation of stride segmentation algorithms on free-living data have been taken, this topic remains an open field in research with the need for optimized and robust methods. In this paper, we make the following contributions to the field of mobile gait analysis: first, we present a comprehensive, free-living dataset of PD patients, including semi-automatically labeled stride borders. Second, a new custom HMM-based stride segmentation approach was developed. The model architecture was chosen to best fit the available training labels as well as to model the expected heterogeneity within free-living gait data. Therefore, two HMMs, one for modelling strides and one for modelling transitions from and to gait, were individually trained and finally combined to a single flattened HMM for segmentation. Configurable model parameters were thoroughly optimized by a grid search to enable the best possible segmentation performance for future applications. Additionally, to test the generalizability of the data-driven HMM-method, model training was performed first only on available laboratory data and second also on the free-living dataset. To estimate the relative performance of the proposed HMMs, their segmentation results were compared to a non-data-driven state-of-the-art DTW-based approach [14], which was initially used to support the manual annotation process of the dataset. Finally, we further investigated the influence of walking bout length on segmentation performance to better understand the limitations and challenges associated with stride segmentation on such free-living datasets. A graphical abstract summarizing this work can be found in the supplementary material (Additional file 1).

## Materials and methods

### Data acquisition

The dataset used for this work was acquired in the FallRiskPD study (DRKS-ID: DRKS00015085) and consists of a cohort of 28 PD patients (Table 1). Inclusion criteria for the study were a diagnosis of Parkinson’s syndrome according to the guideline of the German Society for Neurology (Hoehn and Yahr stage I-III), the ability to walk 4x${10}\hbox { m}$ without support as well as being predominantly ambulatory without walking aids (for the presented population, none of the participants required a walking aid during their daily life). Participants had to be able to read and to understand a set of instructions to operate a wearable sensor-based gait analysis system self-reliantly during a two-week unsupervised free-living phase. Exclusion criteria included , a maximum walking distance of less than ${100}\hbox { m}$, decompensated cardiopulmonary limitations, and other pronounced musculoskeletal disorders that severely limit the ability to move and walk.

The study was approved by the local ethics committee Re-No. 165_18B (Friedrich-Alexander-University Erlangen-Nuremberg, Germany). All patients gave written, informed consent, prior to the data collection, which can be divided into two subsets for this work:

#### At-lab dataset

The patients visited the clinic for a routine checkup and performed standardized gait tests in a laboratory environment and under the supervision of clinical experts. Gait tests like the 4x${10}\hbox { m}$ test (in self-selected, slow and fast speed), a ${2}\hbox { min}$ walk test as well as the timed-up-and-go test were recorded using a mobile gait analysis system.

#### Free-living dataset

After the clinical visit, the patients were equipped with the same mobile gait analysis system and sent home for two weeks for a continuous home-monitoring approach. During this period, the patients wore the sensor system in their wake-time as much as possible (indoors and outdoors), and followed their normal daily activities.

**Table 1:** Patient characteristics (N = 28). Parameters are either given by class or by mean ± standard deviation

Characteristic	
Gender [f/m]	7/21
Age [years]	$63.7 \pm 7.2$
Height [cm]	$174.6 \pm 8.8$
Weight [kg]	$77.1 \pm 15.3$
UPDRS-III	$17.0 \pm 8.1$
Hoehn & Yahr	$2.5 \pm 0.6$

**Fig. 1:**
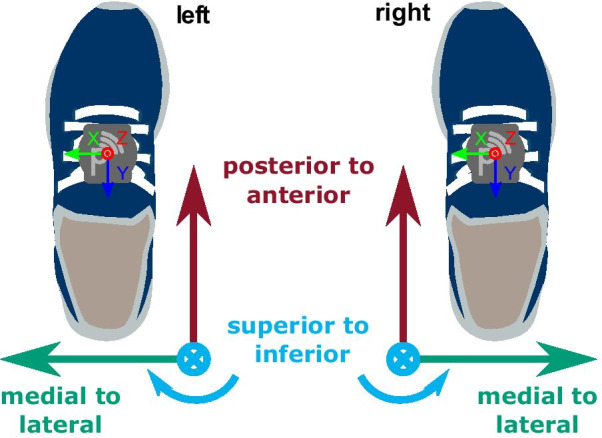
Shoe-/ body-frame coordinate system definition with IMU sensors attached to the instep position of the shoes

All recordings for this study were acquired using the Mobile GaitLab (Portabiles HealthCare Technologies, Erlangen, Germany). The recording system consists of two IMUs (one per foot), attached to the instep position of orthopedic shoes (Fig. 1). Each sensor unit incorporates a 3D-accelerometer (range $\pm 16$ g) and a 3D-gyroscope (range $\pm 1000$ deg/s). The data were recorded at ${102.4}\hbox { Hz}$ and left-right synchronized, such that both sensor units share a common time axis. For the first patients in the study (N = 15), the dataset had to be manually aligned on a bout level, while the rest of the dataset (N = 13) was recorded with an updated version of the Mobile GaitLab featuring hardware synchronization based on a proprietary ${2.4}\hbox { GHz}$ protocol, which was presented in [32]. Both synchronization methods were confirmed to be sufficient for walking bout level definitions by visual inspection. Therefore, all recordings will be handled as a single dataset for this study without any further differentiation between the two utilized hardware versions.

All IMU data were calibrated using the Ferraris method to convert the raw measurement to meaningful physical units of m/s^2^ for the accelerometer and deg/s for the gyroscope [33].

### Coordinate system

To be able to perform stride segmentation on the left and right foot using a single segmentation pipeline, the individual sensor coordinate systems were transformed to a shared body-frame notation with the three main axes defined as medial to lateral (ml), posterior to anterior (pa), and superior to inferior (si) directions (Fig. 1). This coordinate notation will be used throughout the whole manuscript. Additionally, the accelerometer si-axis was aligned to gravity based on static frames within each new walking bout. This ensures a consistent alignment between the sensor- and world frame over the full day recordings and accounts for intra-day coordinate system variations due to the patients putting their shoes on/off as well as attaching/detaching the sensor units.

### Semi-automated stride- and walking bout annotation

To lower the overall annotation effort of the at-lab as well as the free-living dataset, a custom semi-automated labeling tool was used. Therefore, the tool featured an automatic pre-segmentation pipeline consisting of two major steps: First an extraction of activity windows, based on a gyro-norm threshold, and second a pre-segmentation of stride borders within those respective windows. For this purpose, an available DTW-based template matching approach introduced by Barth et al. [14] was used as it did not require any prior training. After applying this first “naïve”, automatic pipeline, each sensor data stream together with the initially generated stride labels was entirely inspected by a human annotator and stride borders were manually added, corrected or deleted wherever necessary. Hence, the overall accuracy of the final reference labels was only dependent on the human annotator performance, this is why a basic yet easy to label stride border definition was chosen.

#### Stride definition

**Fig. 2:**
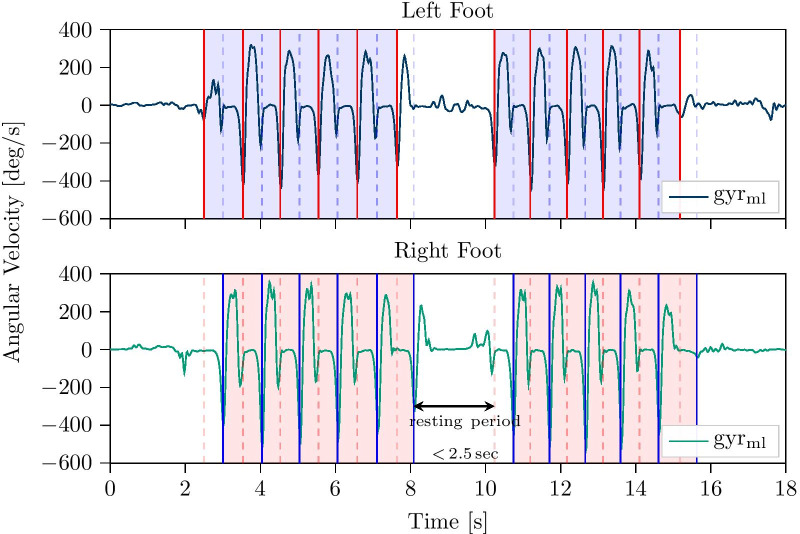
Example data of one walking bout with 20 manually annotated strides and a maximum resting period of ${2.5}\hbox { s}$. Solid lines represent semi-automatically labeled stride borders, while dashed lines correspond to the borders of the opposite foot

The stride definition used for this work was chosen based on previous studies featuring foot-worn IMUs [14, 29], which enabled a consistent manual labeling of clearly visible sensor signal features. A stride was defined based on the angular velocity of the foot in the sagittal plane (gyr$_{\mathrm{ml}}$). The negative peak just before the start of the swing phase was defined as the beginning of a stride, while the negative peak right after the stance phase was defined as its end (Fig. 2).

To achieve consistent labels, the timings of the semi-automated DTW borders, as well as of the manually set borders were corrected by snapping the border to the respective signal minimum within a ${200}\hbox { ms}$ centered window on the gyr$_{\mathrm{ml}}$-axis. The strides were labeled for both the left and the right foot individually. However, the sensor streams were inspected on a shared time axis to be able to identify walking bouts and strides more easily. Manual stride border annotations used within this work were performed by the same single human annotator. A quantitative overview of the number of annotated strides, as well as walking bouts, is given in Table 2. Due to the extensive labeling amount required, the free-living dataset was restricted to one randomly selected day for each subject of the 14 day recording period.

**Table 2:** Number of strides and number of walking bouts per subject (N = 28) for the at-lab as well as the free-living dataset

	N strides		N bouts	
	Mean ± std	Total	Mean ± std	Total
At-lab dataset	$713 \pm 176$	19.964	$13 \pm 3$	379
Free-living dataset	$5234 \pm 2915$	146.574	$189 \pm 84$	5318

#### Walking bout definition

In literature, walking bout definitions range from three steps to more than ${60}\hbox { s}$, based on the used sensor configuration and stride definition [6]. For this work, a walking bout was defined by a minimum number of four strides (based on the synchronized information of both feet) as at least two consecutive strides per foot are required for spatio-temporal parameter calculation using the aforementioned stride border definition. Additionally, a maximum resting period of ${2.5}\hbox { s}$ within a bout was allowed (Fig. 2), as already suggested in other studies [28, 34, 35]. For this work only walking bouts following the given definition were included for the evaluation. Strides outside of walking bouts and non-gait activity windows were not considered.

### Preprocessing and feature extraction

#### Preprocessing

For the segmentation task, the data were low-pass filtered using a fourth-order forward-backward Butterworth filter with a cut-off frequency of ${10}\hbox { Hz}$ to minimize motion noise and at the same time, retain stride border features. Additionally, the data were downsampled by a factor of two to ${51.2}\hbox { Hz}$ using a decimation filter to increase the sample to sample difference and lower the computational effort.

#### Feature extraction

To include additional temporal information for the segmentation process, a sliding, centered window feature extraction was applied, and used as the input for the segmentation model. Windows were shifted by one sample to guarantee a maximum temporal resolution. Features considered for this work where: the raw data itself, the gradient of the linear regression fit, the signal variance, and the second-order polynomial fit, which were partially derived from literature [29]. Finally, z-score standardization was applied per feature axis per predefined walking bout. Different window sizes and feature combinations were tested during the parameter optimization step.

### Hidden Markov Model

To model the sequential nature of human gait, a Hidden Markov Model (HMM) based approach was chosen for this work. An HMM is characterized by a doubly embedded stochastic process [36] of which one stochastic process is described by Markov chains and is referred to as hidden (in this work the sequential human gait model) because it can only be observed through a second stochastic process (the measured IMU data).

**Fig. 3:**
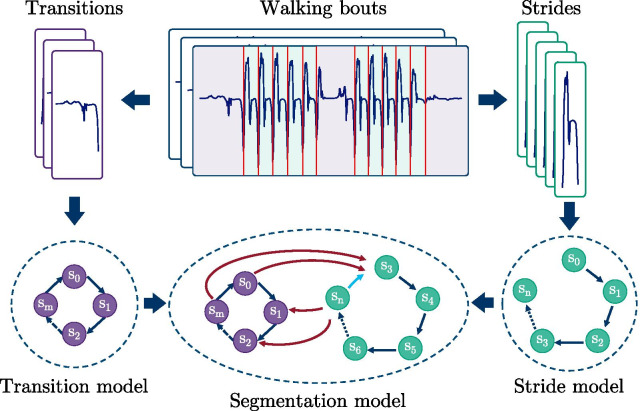
Schematic of a two-class HMM: The transition model and the stride model were trained separately on the respective parts of the training dataset. In a second step, the missing edges were calculated based on the complete training set to connect both models

The proposed HMM architecture was chosen in a way to fit the available data and labels best. As only stride borders were available, the stride segmentation was formulated as a two-class problem, where each class was modeled as an individual HMM: namely, strides, which correspond to the labeled data, as well as transitions, which represent the unlabeled part of the data (but might also include resting). Emission distributions for each HMM were represented using Gaussian mixture models (GMMs) to describe the hidden states. GMMs were chosen to model the expected heterogeneity of different transitions as well as stride characteristics, which are expected in free-living data. HMMs were trained in an unsupervised manner using the Baum-Welch (BW) algorithm, which iteratively refines model parameters using expectation-maximization. BW-training was limited to a maximum of ten iterations, which was found to be sufficient during preliminary testing. To be able to represent the multi-class structure of the given data, the trained models for strides and transitions were finally combined to the actual segmentation model (Fig. 3).

HMMs were implemented using the open source python package pomegranate v0.13.3 [37].

#### Stride Model architecture

**Fig. 4:**
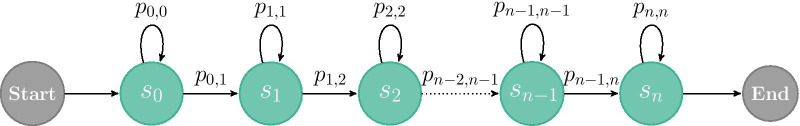
HMM structure of the stride sub-model: A left-right Markov chain from state $s_{0}$ to $s_{n}$, with transition probability $p_{n,n+1}$, and self transition probability $p_{n,n}$. The sequence is enforced to always start with state $s_{0}$ and end with $s_{n}$

Based on the assumption, that a stride must follow a predefined and repetitive bio-mechanical order (swing and stance phase), the stride model was based on a strict left-to-right Markov chain. Each hidden state was linked to a sub-phase of a stride. The time-granularity of those sub-phases was defined by the overall number of states within the model. This means that each stride was represented by a monotonic raising state sequence from $s_0$ to $s_{n}$ with self transition probabilities $p_{n,n}$ and transition probabilities to the adjacent state $p_{n,n+1}$ as illustrated in Fig. 4.

For initialization and training of the stride model, all individual strides (according to the manual stride border annotations) were extracted from each walking bout within the training dataset resulting in *N* training sequences for *N* labeled strides.

Hidden states were initialized by naïvely dividing each stride into *n* equally spaced sections to derive initial parameters for *n* GMMs, with *n* being the number of hidden states. Existing edges within the transition matrix were initialized uniformly. Transition probabilities, as well as, GMM parameters, were then optimized iteratively using the BW-algorithm in an unsupervised manner.

#### Transition Model architecture

**Fig. 5:**
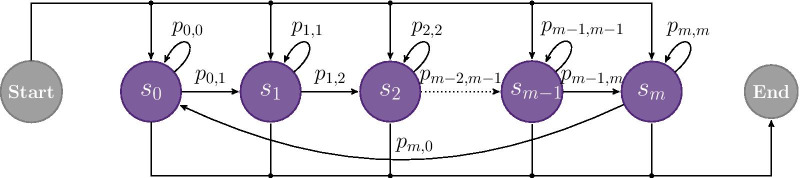
HMM structure of the transition sub-model: A left-right Markov chain from state $s_{0}$ to $s_{m}$, with transition probability $p_{m,m+1}$, and self transition probability $p_{m,m}$. Additionally, a transition from $s_{m}$ to $s_{0}$ was added. Sequences may start and end from any state

Although transitions can also be referred to as a simple “non-stride” or “null-class”, a single hidden state would not be sufficient to model the rather complex nature of transitions to and from gait in free-living data. Therefore, the transition class was modeled as a separate HMM. To account for the fact, that a transition will not follow a strict repetitive bio-mechanical order for a single sequence (compared to a stride), a left-right Markov chain was extended by a transition between the last and the first state. Additionally, a transition sequence is allowed to start and end from any state, resulting in the model architecture as illustrated in Fig. 5.

Parameter initialization and optimization of the transition model was performed in the same way as for the stride model, by naïve linear split and unsupervised BW-training.

The training was performed on *M* transition sequences, where each walking bout of the training dataset included at least two (for start and end of the walking bout) or more transitions. Additional transitions within a valid walking bout were possible following the minimum resting period definition from Section .

#### Model combination

To be able to classify strides and transitions simultaneously and find corresponding stride borders, both individually trained models had to be combined to a single flattened HMM. Therefore, their respective transition matrices were concatenated, so that the resulting matrix was of size (${n+m}\times {n+m}$) with *n* being the number of states of the stride model and *m* the number of states in the transition model. To find the edges, which connect both individual models, first, the hidden state sequence was predicted for each training sequence individually (per stride and per transition using the respective class model). Second, the predicted hidden states were merged to a continuous sequence for each walking bout. Based on this now fully hidden state labeled dataset, all possible state transitions could be derived to update the combined transition matrix. The already learned emission distributions were unchanged as these were already optimized during the previous BW-training.

Model parameters like number of states for the stride- and transition model as well as number of GMM components were optimized during grid search (Table 3).

#### Stride border prediction

**Fig. 6:**
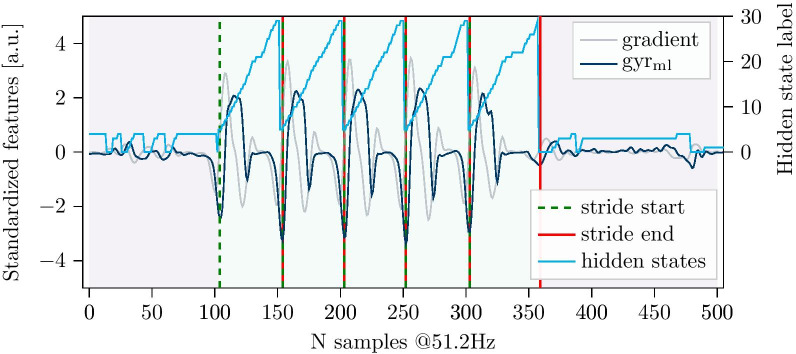
Input signal of a walking bout with predicted hidden state sequence and derived stride borders. States 0 to 4 represent transitions, while states 5 to 29 refer to the stride model

To perform stride segmentation on unseen IMU data (see Fig. 6), first, the most likely hidden state sequence for the respective sensor signal was predicted based on the combined segmentation model using the Viterbi algorithm for inference. Second, any change from a transition state to a stride state or vice versa, plus transitions between the last and first state of the stride sub-model are considered as stride borders. To match the exact stride border definition of the manual labeling process, the borders were set to the minimum value of the gyr$_{\mathrm{ml}}$ axis in the region defined by the two hidden states, between which the transition occurred.

## Evaluation

### Performance metrics

The aim of the stride segmentation was to maximize the number of correctly segmented strides / true positives (TPs) while at the same time the number of missed strides / false negatives (FNs) and the number of falsely detected strides / false positives (FPs) should be minimized. These metrics are commonly represented in terms of precision and recall. As both precision and recall should be maximized for the evaluation of segmentation performance, the F1-score is used, which combines recall and precision by their harmonic mean:

$$\begin{aligned} precision= & {} \frac{TP}{TP + FP} \quad , \quad recall = \frac{TP}{TP + FN} \\ F1= & {} 2 \cdot \frac{precision \cdot recall}{precision + recall} \end{aligned}$$

Because the stride definition used for this study did not correspond to a specific stride event and small variations of stride borders will not have any impact on a following event detection and stride parameter calculation, a stride border was considered valid if it was located within a centered $\pm {60}\hbox { ms}$ window ($\pm 3$ samples) around the ground truth border. Still, a stride was only counted as TP if both, the begin and the end, are within the described margins.

### Evaluation on at-lab data

For baseline evaluation, a 4-fold, nested leave-N-subjects-out cross-validation (CV) was performed on the at-lab dataset. The inner folds were used for grid search and parameter optimization, while the outer folds were used for the performance evaluation. Inner and outer folds were split such that no subject data, which were used during parameter optimization, were used for evaluation to prevent the model from overfitting.

#### HMM grid search

The grid search parameters (Table 3) used for the model training were partly derived from previous literature [16, 29] and partially from pre-study experiments. The sensor axis was fixed to gyr$_{\mathrm{ml}}$ for this study as it contained the main signal features, which were used to define the stride borders.

**Table 3:** Grid search values for HMM hyperparameter optimization. Grad = gradient of the linear regression fit, var = signal variance and poly fit = the first three coefficients of the second-order polynomial fit

Parameters	Values
Window size [ms]	100, 220, 500
Feature combinations	[raw] / [raw, grad] / [raw, var, poly fit]
Number of GMM components	1, 3, 5, 8
Number of states for stride model	5, 10, 15, 20, 25
Number of states for transition model	3, 5, 8, 12

#### DTW grid search

The DTW implementation, which was used for the semi-automated labeling tool, was selected as a state-of-the-art reference method for the proposed HMM. For a fair comparison between the two methods, a parameter grid search was performed for the DTW approach on the at-lab dataset, as well. As for DTW, no actual training step is necessary, the parameter optimization and evaluation were performed only on the outer folds, where the same train-test split as for the HMM evaluation was used. For DTW the maximum warping cost (*max. cost*) threshold was grid searched in a range from 2.0 to 5.0 in steps of 0.25 with the input feature fixed to the raw data of the gyr$_{\mathrm{ml}}$ axis.

For each fold, a new stride template was generated using all strides over all subjects of the train split. For the template generation, each stride was linearly interpolated to the mean number of samples over all strides. Afterwards, the mean per sample over all strides was calculated and used as the template.

### Evaluation on free-living data

For the final evaluation on the free-living dataset, a repeated nested CV including a grid search was omitted due to the high computational demand required for training the model on free-living data. Instead, a leave-one-subject-out CV with a single optimized hyperparameter set was conducted.

#### Hyperparameter selection

To select an optimal hyperparameter set for the HMM training, the mean F1-score per parameter set over all grid search folds (4x4 inner folds) of the previous nested CV on the at-lab dataset was considered. Thus, the parameter set, which reached the on average highest F1-score on the at-lab dataset, was chosen for the free-living evaluation. The optimal *max. cost* threshold for the evaluation of the DTW method on the free-living dataset was directly derived from the at-lab CV as well.

#### HMM training paradigm

To test the generalizability of the proposed HMM, two different training scenarios were applied:

#### At-lab training

Here, the model was trained only on laboratory data recorded during the clinical visit ($\textit{HMM}_{\textit{lab}}$). This training scenario was chosen to test the possibility to transfer a lab-trained model to a free-living context. A laboratory setting is still one of the most commonly used gait analysis settings and often used to develop and evaluate models which shall later be used in unsupervised free-living conditions.

#### Free-living training

In this scenario, the model was trained solely on the free-living dataset ($\textit{HMM}_{\mathrm{free\hbox {-}living}}$). Here, a much higher diversity of different stride types, like initiation-, termination-, or turning-strides, or shuffling-gait is expected within the dataset. As the number of walking bouts and, therefore, the number of strides can vary substantially between subjects for the free-living data, a randomly chosen subset of 50 walking bouts per patient was used for training. This was done to balance the influence of individual subjects on the free-living model.

## Results

### Evaluation on at-lab data

**Table 4:** Results of the stride segmentation performance, in controlled laboratory settings. All values are given as mean ± std over the 4 outer evaluation folds

Method	Precision [%]	Recall [%]	F1-Score [%]
DTW	$94.6 \pm 1.3$	$92.1 \pm 1.4$	$94.6 \pm 1.3$
HMM	$96.1 \pm 1.1$	$96.4 \pm 1.4$	$96.2 \pm 1.1$

Both DTW and HMM achieved promising segmentation results on the at-lab dataset (Table 4), while the HMM achieved a slightly better segmentation performance, reaching a F1-score of ${96.2 \pm 1.1}$ % compared to ${94.6 \pm 1.3}$ % for the DTW approach.

### Evaluation on free-living data

#### Hyperparameter selection

For the HMM method, the optimal hyperparameters set derived from the nested CV were: 8 components for the GMMs, 25 states for the stride model, and 5 states for the transition model, respectively. The best values for feature extraction were a window size of ${220}\hbox { ms}$ as well as a combination of the raw data together with the linear gradient derived from the sliding window view. For DTW, the best performing *max. cost* threshold was 3.5.

#### Evaluation

Compared to the results of the at-lab dataset (Table 4), the segmentation performance on the free-living dataset dropped by almost 10 % for DTW and by 4 % for the HMM (Table 5). To identify significant differences, paired t-tests were performed for statistical analysis. While no relevant difference between the two training paradigms ($\textit{HMM}_{\textit{lab}}$ vs $\textit{HMM}_{\textit{free}\hbox {-}{} \textit{living}}$) could be found, the HMM outperformed the DTW method significantly reaching a maximum F1-score of ${92.4 \pm 4.1}$ % for the $\textit{HMM}_{\textit{free}\hbox {-}{} \textit{living}}$ compared to only ${85.1\pm 9.0}$ % for the DTW approach ($p \le 0.0001$). While the precision is similar for DTW as well as the HMMs, especially the recall value is considerably higher for the $\textit{HMM}_{\textit{free}\hbox {-}{} \textit{living}}$ approach reaching ${95.6 \pm 2.8}$ % compared to only ${83.3 \pm 10. 1}$ % for DTW.

**Table 5:** Results of the stride segmentation performance, for the free-living dataset. All values are given as mean ± std across the 28 subjects, over all strides per day, per patient

Method	Precision [%]	Recall [%]	F1-Score [%]
DTW	$87.2 \pm 8.4$	$83.3 \pm 10.1$	$85.1 \pm 9.0$
$\hbox {HMM}_{\mathrm{lab}}$	$89.4 \pm 5.6$	$95.2 \pm 3.0$	$92.2 \pm 4.2$
$\hbox {HMM}_{\mathrm{free}\hbox {-}\mathrm{living}}$	$89.4 \pm 5.7$	$95.6 \pm 2.8$	$92.4 \pm 4.1$

#### Impact of bout length

Due to the complex nature of free-living data (like the influence of context, environment or disease fluctuations), the drop of segmentation performance between the at-lab recordings and free-living data was further investigated. Therefore, the different bout groups introduced by Del Din et al. [24] were adopted but converted into the respective number of strides. This resulted in the following walking bout subgroups: $4 \le \hbox {N} \le 15$, $15 < \hbox {N} \le 30$, $30 < \hbox {N} \le 50$, $50 < \hbox {N} \le 100$, $100 < \hbox {N} \le 200$, $\hbox {N} > 200$.

**Fig. 7:**
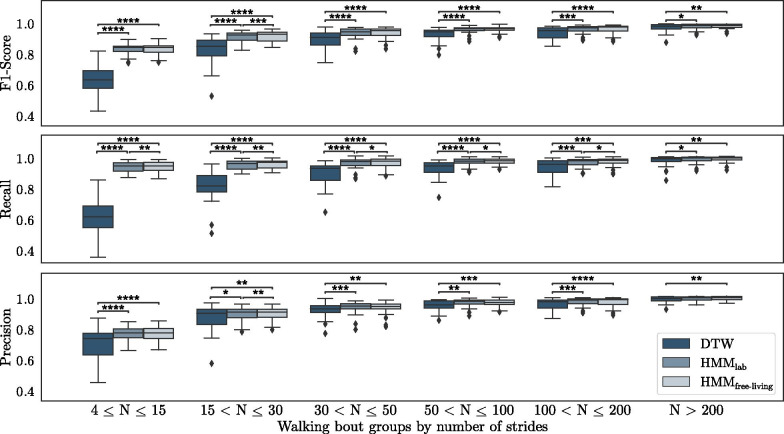
Segmentation performance on the free-living dataset, grouped by the number of strides per walking bout. Significant differences between methods were identified using paired t-tests with *: p $\le$ 0.05, **: p $\le$ 0.01, ***: p $\le$ 0.001 and ****: p $\le$ 0.0001

Performance metrics were re-calculated for each of the defined bout groups per patient and visualized by box plots in Fig. 7. Exact values of performance metrics are summarized in Table 6 as well as in Supplementary Tables S1-S3 (Additional file 2), including respective p-values and Cohen’s d effect sizes. Grouping the segmentation results by the number of strides per bout revealed a relation between segmentation performance and bout length for all methods. The segmentation F1-score for walking bouts $\le 30$ strides was below the overall free-living average, while bouts $\ge 50$ strides were above average and reached comparable performance to the at-lab results for both the HMM as well as the DTW approach.

The HMM-based segmentation approach significantly outperformed the baseline DTW method for all bout groups in terms of precision, recall and F1-score. The biggest difference between DTW and HMM were found within the recall metric over all bout groups. While the performance of all segmentation methods increased with larger bouts, the difference between DTW and HMM became smaller for longer bouts.

#### Impact of training paradigm

Although significant differences between the two training paradigms for the HMM were found in some bout groups, the effect size in terms of Cohen$'$s d was always below 0.2. This indicates only small differences between $\textit{HMM}_{\textit{lab}}$ and $\textit{HMM}_{\textit{free}\hbox {-}{} \textit{living}}$, which is again reflected by the results in Table 6.

**Table 6:** Segmentation performance on the free-living dataset, grouped by the number of strides per walking bout. All values are given as mean ± std

N Strides per bout	Precision [%]			Recall [%]			F1-Score [%]		
	DTW	HMM$_{\mathrm{lab}}$	$\hbox {HMM}_{\mathrm{free}\hbox {-}\mathrm{living}}$	DTW	HMM$_{\mathrm{lab}}$	$\hbox {HMM}_{\mathrm{free}\hbox {-}\mathrm{living}}$	DTW	HMM$_{\mathrm{lab}}$	$\hbox {HMM}_{\mathrm{free}\hbox {-}\mathrm{living}}$
$4 \le \hbox {N} \le 15$	$69.2 \pm 10.2$	$75.7 \pm 4.5$	$75.5 \pm 4.5$	$59.6 \pm 12.4$	$92.7 \pm 3.7$	$93.2 \pm 3.7$	$63.5 \pm 10.2$	$83.3 \pm 3.7$	$83.4 \pm 3.8$
$15 < \hbox {N} \le 30$	$85.5 \pm 8.9$	$88.2 \pm 4.7$	$88.7 \pm 4.7$	$80.5 \pm 10.4$	$94.1 \pm 3.3$	$94.7 \pm 3.3$	$82.8 \pm 9.1$	$91.1 \pm 3.8$	$91.6 \pm 3.5$
$30 < \hbox {N} \le 50$	$90.8 \pm 5.1$	$92.7 \pm 4.2$	$92.6 \pm 4.2$	$88.6 \pm 7.9$	$95.0 \pm 3.7$	$95.5 \pm 3.7$	$89.6 \pm 6.1$	$93.8 \pm 3.7$	$94.0 \pm 3.6$
$50 < \hbox {N} \le 100$	$93.9 \pm 3.8$	$95.7 \pm 2.8$	$95.9 \pm 2.8$	$91.8 \pm 5.7$	$96.2 \pm 2.5$	$96.5 \pm 2.5$	$92.8 \pm 4.6$	$95.9 \pm 2.4$	$96.2 \pm 2.0$
$100 < \hbox {N} \le 200$	$95.1 \pm 3.6$	$96.3 \pm 3.0$	$96.4 \pm 3.0$	$92.8 \pm 5.1$	$95.8 \pm 2.9$	$96.1 \pm 2.9$	$93.9 \pm 4.2$	$96.0 \pm 2.9$	$96.2 \pm 3.0$
$\hbox {N} > 200$	$97.9 \pm 2.2$	$98.4 \pm 1.8$	$98.7 \pm 1.8$	$96.4 \pm 4.0$	$97.4 \pm 2.6$	$97.8 \pm 2.6$	$97.2 \pm 3.0$	$97.9 \pm 2.1$	$98.2 \pm 1.7$

**Fig. 8:**
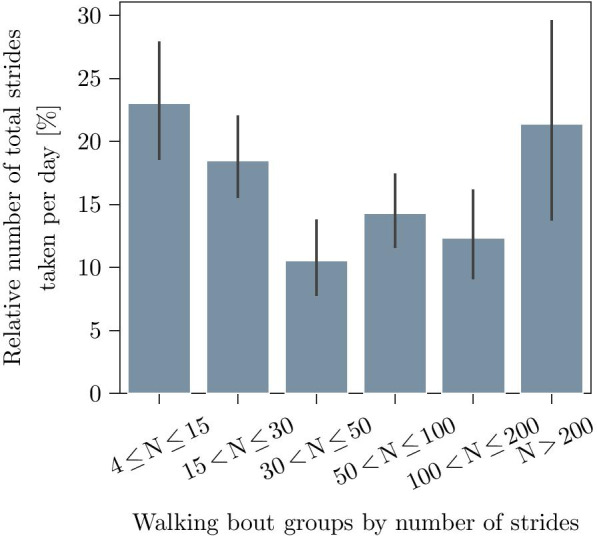
Distribution of the relative number of strides per day for the complete patient cohort. Bars represent the mean and the standard deviation per bout group

## Discussion

To the best of our knowledge, this is the first study, which evaluated stride segmentation performance in foot worn IMU data not only on a large number of 19.964 strides in standardized laboratory settings but also on unsupervised, free-living PD patient data including a total of 146.574 annotated strides. To obtain the required stride borders on both datasets, a semi-automatic manual annotation approach was applied using an existing template matching method based on DTW [14].

Next, a simple yet robust HMM-based segmentation approach was developed. The proposed model was adapted to learn only from manual stride border annotations and best fit the requirements for free-living data analysis. Basic architecture parameters were derived from previous work, where HMM-based stride segmentation was successfully applied on laboratory data [16, 18, 29, 30]. Segmentation performance of the proposed HMM was compared to a state-of-the-art DTW based template matching method [14]. The evaluation of stride segmentation algorithms was continued beyond controlled laboratory settings by validating their performance on free-living PD patient data in a continuous home-monitoring setting. To better understand the impact of the context within free-living gait data, the final evaluation was extended by an in-depth analysis of the impact of bout length on the segmentation performance of the presented methods.

### Performance on laboratory data

To evaluate the baseline performance and find optimal hyperparameters for the presented segmentation methods, a nested CV including an extensive grid search, was conducted on the laboratory dataset. At-lab performance in terms of F1-score was found to be promising for both methods, while the HMM achieved a higher value of ${96.2 \pm 1. 1}$ % compared to the DTW approach performing slightly worse with ${94.6 \pm 1.3}$ %. These results are almost in perfect agreement compared to previous studies in the literature [29]. However, since the at-lab dataset consists of mainly standardized gait test (performed under supervision in clinical hallways), these results cannot be directly transferred to free-living conditions.

### Performance on free-living data

Hence, in the second part of the experiment, the segmentation performance was validated on continuous unsupervised free-living data. Here, two different training paradigms were considered for the HMM method. First, the model was only trained on laboratory data to test the generalizability of the approach as annotated free-living training data might not be available in many cases. Second, the model was trained on actual free-living data as a reference. Additionally, DTW was again applied as a baseline method.

As expected, the segmentation performance was worse for both HMM models as well as for the DTW approach, compared to the performance on the lab dataset. This is most likely related to the increased complexity and heterogeneity of free-living data compared to the controlled laboratory data.

Both HMMs performed almost identical in terms of mean F1-score reaching ${92.2 \pm 4.2}$ % for the $\textit{HMM}_{\textit{lab}}$ and ${92.4 \pm 4.1}$ % for the $\textit{HMM}_{\textit{free}\hbox {-}{} \textit{living}}$, respectively. Although performance dropped by approx. 4 % compared to the laboratory data for the HMM models, they significantly outperformed the reference DTW approach, which only could reach a F1-score of 85.1 % with a high standard deviation of 9.0 %. The lower F1-score of the DTW approach can be explained because of the use of a single stride template, which was not able to cover the expected heterogeneity of strides expected within a free-living dataset. By averaging multiple annotated strides for template generation, information about the variety of different stride types will be lost. Especially non-steady strides will have a high euclidean distance compared to the template resulting in high warping costs. Therefore, such strides were not segmented correctly or at all by the DTW method. For the HMM-based approach, in contrast, the underlying probability distributions of each state of the model were optimized during the training step. Due to the use of GMMs with multiple components per state, the HMM could model and represent a wider variety of different stride types within a single model. This increased complexity and the probabilistic approach make the HMM-based method flexible enough to better handle the heterogeneity of strides within the free-living dataset. Hence, the HMM also correctly segmented unseen and irregular strides resulting in an improved F1-score compared to DTW.

### Impact of bout length

To gain deeper insights into the stride segmentation performance on free-living patient data, results were grouped by previously proposed bout definitions [24]. This revealed a dependency of segmentation performance to bout length for the free-living dataset. Especially for shorter walking bouts with less than 30 strides, F1-scores dropped below average values (reported in Table 5) for all methods, while both HMMs still outperformed DTW significantly. For bouts with more than 50 strides, both, HMM and DTW, achieved a comparable performance as on the at-lab dataset reaching F1-scores of ${96.2 \pm 2.0}$ % up to ${98.2\pm 1.7}$ % for $\textit{HMM}_{\textit{free}\hbox {-}{} \textit{living}}$ and ${92.8\pm 4.5}$ % up to ${97.2\pm 3.0}$ % for DTW.

This relation of segmentation performance and bout length could be explained by an increased percentage of non-steady-strides for shorter bouts. Here, various different stride types like initiation-, termination- or turning-strides as well as shuffling are suspected of generating artifacts or blurring the signal features, which were required to define the stride borders. This could also be confirmed during the manual annotation approach, where stride borders were not always clearly defined particularly for short walking bouts. However, these shorter walking bouts with less than 30, mostly non-steady-strides, make up more than 40 % of total strides taken per day on average for the presented cohort (Fig. 8). This also explains their considerable impact on the overall result. Although the importance of these non-steady strides is still poorly understood within free-living gait data, some studies already showed that this group of strides might yield additional clinical information [38, 39].

In contrast, for longer walking bouts, the proportion of such “non-steady” strides will be lower, while the regularity of strides is expected to rise with increased bout length. This, in turn, would result in a decreased complexity of the underlying IMU signals with clear stride border features and hence explain the better segmentation performance.

The overall F1-score performance of the HMM-based segmentation was dominated by its recall values, which yield significant differences between HMM and DTW up to bouts with $\le 200$ strides, while the precision was only noticeably different for the first bout group with $< 15$ strides.

This could be explained by the fact that a two-class model for the HMM method might not capture the entire complexity of free-living data and hence the HMM switches into its stride sub-model too often. However, such false positives might be easily eliminated by a subsequent post-processing step like an event detection. Therefore, methods presented by Rampp et al. [40] could be used for a detection of heel-strike, toe-off, and mid-stance to validate a segmented stride candidate, which should boost the overall precision, while preserving the already high recall.

### Impact of training paradigm

For the overall F1-score no significant difference between the two models $\textit{HMM}_{\textit{free}\hbox {-}{} \textit{living}}$ and $\textit{HMM}_{\textit{lab}}$ could be found. Although the $\textit{HMM}_{\textit{free}\hbox {-}{} \textit{living}}$ achieved a significantly better recall value compared to the $\textit{HMM}_{\textit{lab}}$, the absolute difference between the two training paradigms was always below the standard deviation with Cohen’s d values $< 0.2$ for all bout groups. The major disadvantage of training the HMM on free-living data is the extensive amount of manual labeling and verification, which was necessary to generate the required ground truth labels. In contrast, the $\textit{HMM}_{\textit{lab}}$ has the advantage that reference stride borders required for training can be obtained easier and with less effort, for example, during routine clinical checkups. Therefore, the presented results might indicate a reasonable generalizability of the proposed HMM with the flexibility to handle completely unseen data from a different context due to its probabilistic approach. Thus, a lab trained model could be directly transferred to the free-living domain with some additional improvements regarding the precision using post-processing steps as mentioned before.

### Limitations

Generating reference labels on completely unsupervised free-living data is still an open and unsolved challenge in IMU-based gait analysis. In this work, we tried to address this problem by a time costly manual annotation approach. As no alternative gold-standard reference system was available, annotation had to be performed directly on the IMU time-series signal. Although foot-mounted IMUs have the advantage of a high biomechanical resolution, stride borders were not always completely clear and, therefore, dependent on the subjective assessment of the annotator. Hence, the presence of isolated human annotation errors cannot be avoided entirely in the used reference labels. However, due to a large number of almost 150.000 annotated strides, the influence of human annotation errors should be on a negligible scale for the primary outcome of this work.

Furthermore, only predefined walking bouts were considered for the evaluation within this work. Walking bouts were derived from the manual stride border annotations, to avoid potential biases due to additional pre-processing steps (which will require individual evaluation in the future). This means that the presented segmentation model will be dependent on a robust gait sequence detection as a preceding step to be used for continuous free-living data. Methods based on the analysis of harmonic frequencies, as presented by Ullrich et al. [41], could solve this problem.

## Conclusion

For this work, a comprehensive evaluation dataset for free-living stride segmentation was presented, including 146.574 semi-automated labeled strides of 28 PD patients. A custom HMM-based stride segmentation approach was introduced and evaluated together with an available state-of-the-art DTW-based reference method on laboratory as well as free-living data. The proposed HMM method was able to outperform the DTW-based approach on both datasets significantly. Further investigations revealed a relation between walking bout length and segmentation F1-score. Especially bouts with $\le 30$ strides showed a reduced performance below average, while bouts with $> 50$ strides reached a similar performance compared to results in the laboratory setting. Using free-living data compared to lab data for training did not improve the overall segmentation performance noticeably, which could indicate a good generalizability of the model. The HMM demonstrated its strength, especially for the recall metric, while the precision needs further improvement. This issue might be solved by additional post-processing steps to reliably identify false positive strides, for example, by a subsequent event detection or a simple stride-feature based classifier.

Although the presented HMM could improve segmentation performance significantly, compared to the DTW baseline approach, especially the shorter and more challenging walking bouts will require further technical attention. For this purpose, deep learning models based on convolutional neural networks (CNNs) [21, 22] or recently proposed recurrent neural networks (RNNs) [42] might be able to improve segmentation performance even further and help to model the expected heterogeneity within those specific bout groups. Hence, in future work, additional segmentation benchmarks should be carried out on free-living gait datasets to identify other suitable stride segmentation methods for upcoming applications in the field of continuous free-living mobile gait analysis.

Nevertheless, our proposed probabilistic HMM-based stride segmentation approach proved to be a promising candidate to handle the increased heterogeneity and complexity within free-living gait. The presented results highlight the challenges of stride segmentation on free-living datasets compared to controlled laboratory assessments as well as the influence of walking bout length on respective segmentation performance. Also, environmental factors like underground or the number of corners during unsupervised free-living studies might affect gait patterns and therefore require robust segmentation methods with respective evaluations. Therefore, our presented work is an important step towards a robust and reliable assessment of stride parameters for future free-living gait analysis applications and respective clinical insights into neurological diseases.

## Supplementary Information

**Additional file 1.** Flow chart summarizing the presented work.

**Additional file 2: Table S1–S3.** Detailed performance metrics of the free-living segmentation evaluation together with respective p-values and Cohen’s d effect sizes.

## Data Availability

The datasets used and/or analyzed during the current study are available from the corresponding author on reasonable request and after approval by the ethical committee in case patient related data is requested.

## Abbreviations

CV: Cross validation
DTW: Dynamic time warping
FN: False negative
FP: False positive
HMM: Hidden Markov Model
HSP: Hereditary spastic paraplegia
IMU: Inertial measurement unit
ML: Medial to lateral
PD: Parkinson’s disease
TP: True positive

## References

[1] Mirelman A, Bonato P, Camicioli R, Ellis TD, Giladi N, Hamilton JL, Hass CJ, Hausdorff JM, Pelosin E, Almeida QJ (2019). Gait impairments in Parkinson’s disease. Lancet Neurol.

[2] Bortone I, Buongiorno D, Lelli G, Di Candia A, Cascarano GD, Trotta GF, Fiore P, Bevilacqua V. Gait analysis and parkinson’s disease: Recent trends on main applications in healthcare. In: International Conference on NeuroRehabilitation, Springer; 2018. p. 1121–1125.

[3] Brognara L, Palumbo P, Grimm B, Palmerini L (2019). Assessing gait in parkinson’s disease using wearable motion sensors: a systematic review. Diseases.

[4] Schlachetzki JC, Barth J, Marxreiter F, Gossler J, Kohl Z, Reinfelder S, Gassner H, Aminian K, Eskofier BM, Winkler J (2017). Wearable sensors objectively measure gait parameters in Parkinson’s disease. PLoS ONE.

[5] Sica M, Tedesco S, Crowe C, Kenny L, Moore K, Timmons S, Barton J, O’Flynn B, Komaris D-S (2021). Continuous home monitoring of parkinson’s disease using inertial sensors: a systematic review. PLoS ONE.

[6] Del Din S, Godfrey A, Mazzà C, Lord S, Rochester L (2016). Free-living monitoring of Parkinson’s disease: lessons from the field. Movement Disorders.

[7] Bobić VN, Djurić-Jovièić MD, Radovanović SM, Dragaević NT, Kostić VS, Popović MB. Challenges of stride segmentation and their implementation for impaired gait. In: 2018 40th Annual International Conference of the IEEE Engineering in Medicine and Biology Society (EMBC), IEEE; 2018. p. 2284–2287. 10.1109/EMBC.2018.8512836 30440862

[8] Lueken M, ten Kate W, Batista JP, Ngo C, Bollheimer C, Leonhardt S. Peak detection algorithm for gait segmentation in long-term monitoring for stride time estimation using inertial measurement sensors. In: 2019 IEEE EMBS International Conference on Biomedical & Health Informatics (BHI), IEEE; 2019 pp. 1–4.

[9] Lueken M, Warner RT, Kate T, Valenti G, Batista J, Bollheimer C, Leonhardt S, Ngo C. Estimation of stride time variability in unobtrusive long-term monitoring using inertial measurement sensors. IEEE J Biomed Health Inform. 2020. 10.1109/JBHI.2020.2992448 32386168

[10] McCamley J, Donati M, Grimpampi E, Mazza C (2012). An enhanced estimate of initial contact and final contact instants of time using lower trunk inertial sensor data. Gait & Posture.

[11] Boutaayamou M, Brüls O, Denoël V, Schwartz C, Demonceau M, Garraux G, Verly JG. Segmentation of gait cycles using foot-mounted 3D accelerometers. In: 2015 International Conference on 3D Imaging (IC3D), IEEE; 2015. p. 1–7.

[12] Boutaayamou M, Denoël V, Brüls O, Demonceau M, Maquet D, Forthomme B, Croisier J-L, Schwartz C, Verly JG, Garraux G. Algorithm for temporal gait analysis using wireless foot-mounted accelerometers. In: International Joint Conference on Biomedical Engineering Systems and Technologies, Springer; 2016. p. 236–254.

[13] Prateek G, Mazzoni P, Earhart GM, Nehorai A. Gait cycle validation and segmentation using inertial sensors. IEEE Trans Biomed Eng. 2019. 10.1109/TBME.2019.2955423 PMC7305840 31765301

[14] Barth J, Oberndorfer C, Pasluosta C, Schülein S, Gassner H, Reinfelder S, Kugler P, Schuldhaus D, Winkler J, Klucken J (2015). Stride segmentation during free walk movements using multi-dimensional subsequence dynamic time warping on inertial sensor data. Sensors.

[15] Vienne-Jumeau A, Oudre L, Moreau A, Quijoux F, Edmond S, Dandrieux M, Legendre E, Vidal PP, Ricard D (2020). Personalized template-based step detection from inertial measurement units signals in multiple sclerosis. Front Neurol.

[16] Pfau T, Ferrari M, Parsons K, Wilson A (2008). A hidden Markov model-based stride segmentation technique applied to equine inertial sensor trunk movement data. J Biomech.

[17] Mannini A, Sabatini AM. A hidden Markov model-based technique for gait segmentation using a foot-mounted gyroscope. In: 2011 Annual International Conference of the IEEE Engineering in Medicine and Biology Society (EMBC), IEEE; 2011. p. 4369–4373. 10.1109/IEMBS.2011.6091084 22255307

[18] Mannini A, Sabatini AM (2012). Gait phase detection and discrimination between walking-jogging activities using hidden Markov models applied to foot motion data from a gyroscope. Gait & Posture.

[19] Mannini A, Trojaniello D, Della Croce U, Sabatini AM. Hidden Markov model-based strategy for gait segmentation using inertial sensors: application to elderly, hemiparetic patients and Huntington’s disease patients. In: 2015 37th Annual International Conference of the IEEE Engineering in Medicine and Biology Society (EMBC), IEEE; 2015. p. 5179–5182. 10.1109/EMBC.2015.7319558 26737458

[20] Martindale CF, Sprager S, Eskofier BM (2019). Hidden Markov model-based smart annotation for benchmark cyclic activity recognition database using wearables. Sensors.

[21] Gadaleta M, Cisotto G, Rossi M, Rehman RZU, Rochester L, Del Din S. Deep learning techniques for improving digital gait segmentation. In: 2019 41st Annual International Conference of the IEEE Engineering in Medicine and Biology Society (EMBC), IEEE; 2019. p. 1834–1837. 10.1109/EMBC.2019.8856685 31946254

[22] Steinmetzer T, Bönninger I, Reckhardt M, Reinhardt F, Erk D, Travieso CM. Comparison of algorithms and classifiers for stride detection using wearables. Neural Comput Appl. 2019;1–12.

[23] Ottosson J, Lavesson L, Pinzke S, Grahn P (2015). The significance of experiences of nature for people with Parkinson’s disease, with special focus on freezing of gait—the necessity for a biophilic environment. A multi-method single subject study. Int J Environ Res Public Health.

[24] Del Din S, Godfrey A, Galna B, Lord S, Rochester L (2016). Free-living gait characteristics in ageing and Parkinson’s disease: impact of environment and ambulatory bout length. J NeuroEng Rehabil.

[25] Tamburini P, Storm F, Buckley C, Bisi MC, Stagni R, Mazzà C (2018). Moving from laboratory to real life conditions: influence on the assessment of variability and stability of gait. Gait & Posture.

[26] Storm FA, Nair K, Clarke AJ, Van der Meulen JM, Mazzà C (2018). Free-living and laboratory gait characteristics in patients with multiple sclerosis. PLoS ONE.

[27] Zhao H, Wang Z, Qiu S, Wang J, Xu F, Wang Z, Shen Y (2019). Adaptive gait detection based on foot-mounted inertial sensors and multi-sensor fusion. Inform Fusion.

[28] Hickey A, Del Din S, Rochester L, Godfrey A (2016). Detecting free-living steps and walking bouts: validating an algorithm for macro gait analysis. Physiol Meas.

[29] Haji Ghassemi N, Hannink J, Martindale CF, Gaßner H, Müller M, Klucken J, Eskofier BM (2018). Segmentation of gait sequences in sensor-based movement analysis: a comparison of methods in Parkinson’s disease. Sensors.

[30] Martindale CF, Roth N, Gaßner H, List J, Regensburger M, Eskofier BM, Kohl Z (2019). Technical validation of an automated mobile gait analysis system for hereditary spastic paraplegia patients. IEEE J Biomed Health Inform.

[31] Panahandeh G, Mohammadiha N, Leijon A, Händel P (2013). Continuous hidden Markov model for pedestrian activity classification and gait analysis. IEEE Trans Instrum Meas.

[32] Roth N, Martindale CF, Eskofier BM, Gaßner H, Kohl Z, Klucken J (2018). Synchronized sensor insoles for clinical gait analysis in home-monitoring applications. Curr Direct Biomed Eng.

[33] Ferraris F, Grimaldi U, Parvis M (1995). Procedure for effortless in-field calibration of three-axis rate gyros and accelerometers. Sens Mater.

[34] Aminian K, Najafi B, Büla C, Leyvraz P-F, Robert P (2002). Spatio-temporal parameters of gait measured by an ambulatory system using miniature gyroscopes. J Biomech.

[35] Del Din S, Galna B, Godfrey A, Bekkers EM, Pelosin E, Nieuwhof F, Mirelman A, Hausdorff JM, Rochester L (2019). Analysis of free-living gait in older adults with and without Parkinson’s disease and with and without a history of falls: identifying generic and disease-specific characteristics. J Gerontol.

[36] Rabiner L, Juang B (1986). An introduction to hidden Markov models. IEEE ASSP Magaz.

[37] Schreiber J (2017). Pomegranate: fast and flexible probabilistic modeling in python. J Mach Learn Res.

[38] Nguyen A, Roth N, Ghassemi NH, Hannink J, Seel T, Klucken J, Gassner H, Eskofier BM (2019). Development and clinical validation of inertial sensor-based gait-clustering methods in Parkinson’s disease. J NeuroEng Rehabil.

[39] Haji Ghassemi N, Hannink J, Roth N, Gaßner H, Marxreiter F, Klucken J, Eskofier BM (2019). Turning analysis during standardized test using on-shoe wearable sensors in Parkinson’s disease. Sensors.

[40] Rampp A, Barth J, Schülein S, Gaßmann K-G, Klucken J, Eskofier BM (2014). Inertial sensor-based stride parameter calculation from gait sequences in geriatric patients. IEEE Trans Biomed Eng.

[41] Ullrich M, Küderle A, Hannink J, Del Din S, Gassner H, Marxreiter F, Klucken J, Eskofier BM, Kluge F. Detection of gait from continuous inertial sensor data using harmonic frequencies. IEEE J Biomed Health Inform. 2020. 10.1109/JBHI.2020.2975361 32086225

[42] Martindale CF, Christlein V, Klumpp P, Eskofier BM. Wearables-based multi-task gait and activity segmentation using recurrent neural networks. Neurocomputing. 2020.

