# Supplementary material for: Hidden Markov Model based stride segmentation on unsupervised free-living gait data in Parkinson’s disease patients
Source: J Neuroeng Rehabil. 2021 Jun 3;18:93. doi: 10.1186/s12984-021-00883-7 (PMC8173987; doi:10.1186/s12984-021-00883-7)
Supplement: Supplementary file 1 — Additional file 1. Flow chart summarizing the presented work. [file 12984_2021_883_MOESM1_ESM.pdf]

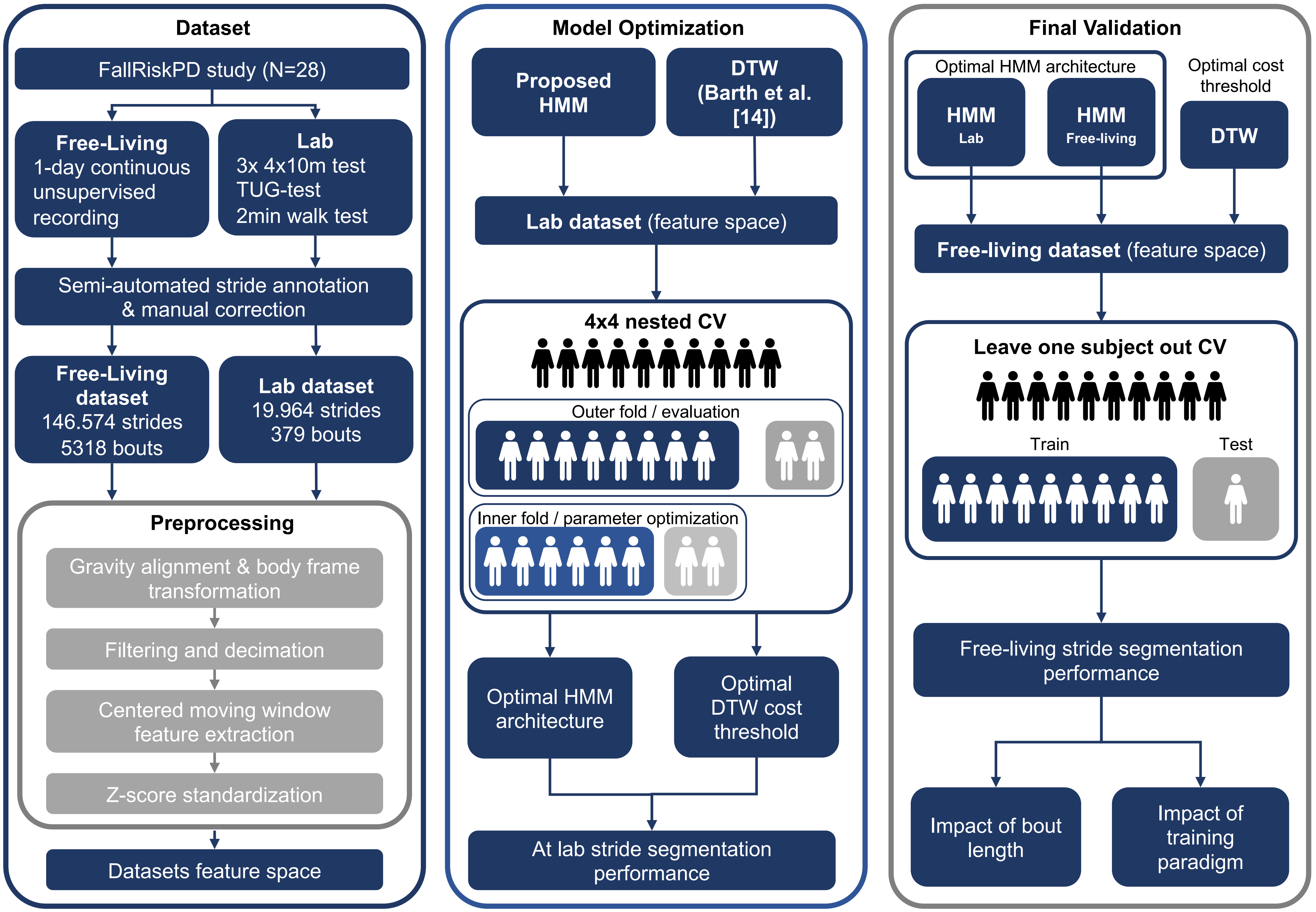

Dataset
FallRiskPD study (N=28)
Free-Living
1-day continuous unsupervised recording
Lab
3x 4x10m test
TUG-test
2min walk test
Semi-automated stride annotation & manual correction
Free-Living dataset
146.574 strides
5318 bouts
Lab dataset
19.964 strides
379 bouts
Preprocessing
Gravity alignment & body frame transformation
Filtering and decimation
Centered moving window feature extraction
Z-score standardization
Datasets feature space
Model Optimization
Proposed HMM
DTW (Barth et al. [14])
Lab dataset (feature space)
4x4 nested CV
Outer fold / evaluation
Inner fold / parameter optimization
Optimal HMM architecture
Optimal DTW cost threshold
At lab stride segmentation performance
Final Validation
Optimal HMM architecture
HMM
Lab
HMM
Free-living
Optimal cost threshold
DTW
Free-living dataset (feature space)
Leave one subject out CV
Train
Test
Free-living stride segmentation performance
Impact of bout length
Impact of training paradigm
