# Supplementary material for: Hidden Markov Model based stride segmentation on unsupervised free-living gait data in Parkinson’s disease patients
Source: J Neuroeng Rehabil. 2021 Jun 3;18:93. doi: 10.1186/s12984-021-00883-7 (PMC8173987; doi:10.1186/s12984-021-00883-7)
Supplement: Supplementary file 2 — Additional file 2: Table S1–S3. Detailed performance metrics of the free-living segmentation evaluation together with respective p-values and Cohen’s d effect sizes. [file 12984_2021_883_MOESM2_ESM.pdf]

Supplementary Table S1: DTW vs HMM_{lab}: Segmentation performance on the free-living dataset, grouped by the number of strides per walking bout. All values are given as mean ± std. P values <0.01 depicted in bold.

**N Strides per bout**	**Precision** [%]				**Recall** [%]				**F1-Score** [%]			
	DTW	HMM_{lab}	p	Cohen's d	DTW	HMM_{lab}	p	Cohen's d	DTW	HMM_{lab}	p	Cohen's d
$4 \leq N \leq 15$	69.2 ± 10.2	75.7 ± 4.5	**<0.001**	0.828	59.6 ± 12.4	92.7 ± 3.7	**<0.001**	3.619	63.5 ± 10.2	83.3 ± 3.7	**<0.001**	2.579
$15 < N \leq 30$	85.5 ± 8.9	88.2 ± 4.7	**0.017**	0.387	80.5 ± 10.4	94.1 ± 3.3	**<0.001**	1.768	82.8 ± 9.1	91.1 ± 3.8	**<0.001**	1.195
$30 < N \leq 50$	90.8 ± 5.1	92.7 ± 4.2	**0.001**	0.405	88.6 ± 7.9	95.0 ± 3.7	**<0.001**	1.036	89.6 ± 6.1	93.8 ± 3.7	**<0.001**	0.835
$50 < N \leq 100$	93.9 ± 3.8	95.7 ± 2.8	**0.001**	0.549	91.8 ± 5.7	96.2 ± 2.5	**<0.001**	1.003	92.8 ± 4.6	95.9 ± 2.4	**<0.001**	0.868
$100 < N \leq 200$	95.1 ± 3.6	96.3 ± 3.0	**<0.001**	0.380	92.8 ± 5.1	95.8 ± 2.9	**0.001**	0.703	93.9 ± 4.2	96.0 ± 2.9	**<0.001**	0.589
$N > 200$	97.9 ± 2.2	98.4 ± 1.8	0.057	0.242	96.4 ± 4.0	97.4 ± 2.6	0.039	0.288	97.2 ± 3.0	97.9 ± 2.1	0.036	0.291

Supplementary Table S2: DTW vs $\text{HMM}_{\text{free-living}}$: Segmentation performance on the free-living dataset, grouped by the number of strides per walking bout. All values are given as mean ± std. P values <0.01 depicted in bold.

**N Strides per bout**	**Precision** [%]				**Recall** [%]				**F1-Score** [%]			
	DTW	$\text{HMM}_{\text{free-living}}$	p	Cohen's d	DTW	$\text{HMM}_{\text{free-living}}$	p	Cohen's d	DTW	$\text{HMM}_{\text{free-living}}$	p	Cohen's d
$4 \leq N \leq 15$	69.2 ± 10.2	75.5 ± 4.5	**<0.001**	0.795	59.6 ± 12.4	93.2 ± 3.7	**<0.001**	3.700	63.5 ± 10.2	83.4 ± 3.8	**<0.001**	2.582
$15 < N \leq 30$	85.5 ± 8.9	88.7 ± 4.7	**0.007**	0.452	80.5 ± 10.4	94.7 ± 3.3	**<0.001**	1.859	82.8 ± 9.1	91.6 ± 3.5	**<0.001**	1.281
$30 < N \leq 50$	90.8 ± 5.1	92.6 ± 4.2	**0.001**	0.381	88.6 ± 7.9	95.5 ± 3.7	**<0.001**	1.132	89.6 ± 6.1	94.0 ± 3.6	**<0.001**	0.881
$50 < N \leq 100$	93.9 ± 3.8	95.9 ± 2.8	**0.001**	0.610	91.8 ± 5.7	96.5 ± 2.5	**<0.001**	1.112	92.8 ± 4.6	96.2 ± 2.0	**<0.001**	0.967
$100 < N \leq 200$	95.1 ± 3.6	96.4 ± 3.0	**<0.001**	0.376	92.8 ± 5.1	96.1 ± 2.9	**<0.001**	0.772	93.9 ± 4.2	96.2 ± 3.0	**<0.001**	0.625
$N > 200$	97.9 ± 2.2	98.7 ± 1.8	**0.005**	0.437	96.4 ± 4.0	97.8 ± 2.6	**0.005**	0.404	97.2 ± 3.0	98.2 ± 1.7	**0.004**	0.446

Supplementary Table S3: HMM_{lab} vs $\text{HMM}_{\text{free-living}}$: Segmentation performance on the free-living dataset, grouped by the number of strides per walking bout. All values are given as mean ± std. P values <0.01 depicted in bold.

**N Strides per bout**	**Precision** [%]				**Recall** [%]				**F1-Score** [%]			
	HMM_{lab}	$\text{HMM}_{\text{free-living}}$	p	Cohen's d	HMM_{lab}	$\text{HMM}_{\text{free-living}}$	p	Cohen's d	HMM_{lab}	$\text{HMM}_{\text{free-living}}$	p	Cohen's d
$4 \leq N \leq 15$	75.7 ± 4.5	75.5 ± 4.5	0.276	-0.042	92.7 ± 3.7	93.2 ± 3.7	**0.005**	0.152	83.3 ± 3.7	83.4 ± 3.8	0.541	0.025
$15 < N \leq 30$	88.2 ± 4.7	88.7 ± 4.7	**0.005**	0.095	94.1 ± 3.3	94.7 ± 3.3	**0.001**	0.181	91.1 ± 3.8	91.6 ± 3.5	**0.001**	0.134
$30 < N \leq 50$	92.7 ± 4.2	92.6 ± 4.2	0.683	-0.022	95.0 ± 3.7	95.5 ± 3.7	0.041	0.137	93.8 ± 3.7	94.0 ± 3.6	0.385	0.050
$50 < N \leq 100$	95.7 ± 2.8	95.9 ± 2.8	0.353	0.052	96.2 ± 2.5	96.5 ± 2.5	0.037	0.147	95.9 ± 2.4	96.2 ± 2.0	0.090	0.107
$100 < N \leq 200$	96.3 ± 3.0	96.4 ± 3.0	0.895	0.006	95.8 ± 2.9	96.1 ± 2.9	0.039	0.108	96.0 ± 2.9	96.2 ± 3.0	0.201	0.058
$N > 200$	98.4 ± 1.8	98.7 ± 1.8	0.108	0.191	97.4 ± 2.6	97.8 ± 2.6	0.063	0.142	97.9 ± 2.1	98.2 ± 1.7	0.077	0.175
